# Quantitative data on plant macrofossil distribution in the holocene sediment cores of mires in the Kaliningrad region, Russian Federation (South-Eastern Baltic)

**DOI:** 10.1016/j.dib.2019.104138

**Published:** 2019-06-19

**Authors:** Maxim Napreenko, Tatiana Napreenko-Dorokhova

**Affiliations:** a Shirshov Institute of Oceanology, Russian Academy of Sciences, 36 Nahimovskiy Prospekt, 117997, Moscow, Russia; b Immanuel Kant Baltic Federal University, 14 Nevskogo Street, 236041, Kaliningrad, Kaliningrad region, Russia

**Keywords:** Holocene sediment cores, Micropaleontology, Plant macro-remnants, Mires, Baltic region

## Abstract

The data file presents information on the quantitative taxa distribution for plant macrofossils in the Holocene sediment cores of mires in the Kaliningrad region (Russian Federation, South-Eastern Baltic Region), as well as 24 radiocarbon dates (14C). The dataset contains percentages of 51 most common (more than 1%) taxa in 1361 samples of 45 sediment cores. These data provide information on environmental development and evolution of the azonal wetland vegetation in the south-eastern part of the Baltic region during the Holocene.

**Table:** Specifications table

Subject area	*Earth Sciences*
More specific subject area	Palaeobotany
Type of data	*Table*
How data was acquired	*Microscope (Olympus CX33, Micromed 3–20, Altami CMO-0745), liquid scintillation spectrometer (1220 Quantulus)*
Data format	*Raw*
Experimental factors	*Data were obtained using a standard laboratory treatment of the peat sediment samples. The samples of wet peat were cleaned under running water. The cleaned material was retained on a* 250 μm *sieve in case of slightly decomposed peat and on a* 100 μm *sieve at a high degree of decomposition.*
Experimental features	*In most cases, four standard microscope slides were examined for each sample. For some specimens, methylene blue was dropped to stain plant tissues in order for more precise identification of plant taxa.*
Data source location	*Shirshov Institute of Oceanology*, Russian Academy of Sciences*, Moscow, Russia*
Data accessibility	*Data are presented with this article*

**Table:** 

**Value of the data** • The published data on the quantitative distribution of different plant macro-remnants in peat cores provide information on vegetation successions in mire ecosystems in the South-Eastern Baltic region during certain periods of the Holocene. • Our palaeoenvironmental data are obtained according to the standard technique so that they can be used without significant modification by other specialists in palaeogeography, palaeobotany and allied spheres of the Earth science. • Main research output from the use of our datasets is a reconstruction of palaeoenvironments in the south-eastern part of the Baltic region during the Holocene. • Our data can be integrated into more wide-scale investigations of the environment and climate development during different periods of the Holocene. • In some cases, these palaeoenvironmental data could be considered as proxies for identification of a transgression event impact onto coastal ecosystems in the South-Eastern Baltic.

## 1 Data

**Fig. 1:**
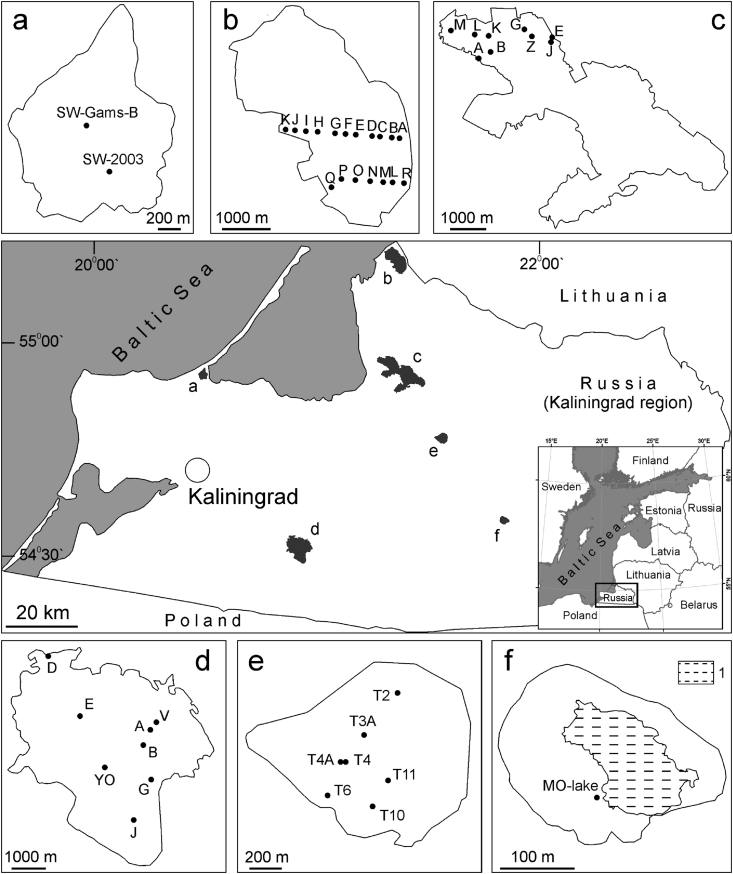
Location of the sediment cores which are provided with data files: a) Svinoye raised bog, b) Kozye raised bog, c) Bolshoye Mokhovoye raised bog, d) Zehlau raised bog, e) Vittgirrenskoye peatland, f) Maloye Olenye terrestrializing lake 1 – unterrestrialized part of Maloye Olenye lake.

The data file contains the quantitative information on relative content (%) of the abundant (>1% of concentration) plant macro-remnants (identified to species or genus) the in the Holocene sediment cores from peatlands in the Kaliningrad region. For certain horizons, the radiocarbon data are provided. The plant macro-remnants is a basic substrate of the peat sediments which have generally no hiatuses in sedimentation and may have reliably been dated by the ^14^С method. This enables to obtain data on environment and climate development with a high time resolution. We provide data for 45 peat sediment cores retrieved from 6 mire ecosystems in the Kaliningrad Region (Fig. 1; Table 1). The data were collected during 2007–2018.

**Table 1:** Location of sediment cores.

Mire	Number of boreholes	Core length, m (min-max)	Area
Svinoye raised bog	2	6.4–7.5	Heavily drained raised bog occupying a proximal part of the offshore bar (Curonian Spit) between the Baltic Sea coast and the Curonian Lagoon N 54°57.80′ E 20°30.96′
Kozye raised bog	18	1.5–7.0	Nearly natural raised bog in deltaic lowland landscape (Neman Delta area), 7 km from the Curonian Lagoon coast N 55°14.96′ E 21°23.80′
Bolshoye Mokhovoye raised bog	9	5.8–11.0	Nearly natural raised bog in deltaic lowland landscape (southern Neman Delta area), 9 km from the Curonian Lagoon coast N 54°58.24′ E 21°22.81′
Zehlau raised bog	8	2.15–5.85	Nearly natural raised bog on a glaciolacustrine plain, 55 km inland from the Baltic Sea coast N 54°31.89′ E 20°55.01′
Vittgirrenskoye peatland	7	0.3–1.1	A morainic plain peatland abandoned after peat extraction, 70 km inland from the Baltic Sea coast N 54°47.98′ E 21°39.47′
Maloye Olenye terrestrializing lake	1	3.4	Pristine transition mire on a buoyant mat developing inward a lake on a glaciolacustrine plain, 85 km inland from the Baltic Sea coast N 54°34.01′ E 21°42.15′

## 2 Experimental design, materials, and methods

The peat bed coring and peat samples retrieval were carried out by means of the Russian D-corer (model TBG-1) taking into account standard methodological guidelines [1]. The laboratory treatment of sediment samples and specimen preparation were executed according to the standard technique [2], [3]: in order to remove non-structured humus particles, a sample of wet peat was cleaned under running water, and the washed material was retained on a 250 μm sieve in case of slightly decomposed peat and on a 100 μm sieve at a high degree of decomposition. For the microscopic analysis, a specimen was prepared in the following way: a small amount of the washed peat has been placed on a microscope slide, several drops of water were added in order to spread the plant residues equally over the slide with a thin layer. The specimen was studied under the microscope which included both identifications of plant macro-remnants and estimation of percentage for different systematic groups of plants in a peat sample. No less than 4 standard microscope slides were examined for each peat sample. A number of atlases and taxonomic keys were used for identification of a systematic group of the plant residues [2], [4], [5], [6], [7]. In some cases, plant residues were stained with methylene blue for identification purposes, in particular, for more distinct detection of pore shape and pore arrangement throughout a leaf.

The radiocarbon analysis of peat and gyttja samples was performed by conventional (14C) dating at the Radiocarbon Laboratory of the Institute of Geography of Russian Academy of Sciences (Moscow, Russia). All radiocarbon dates were calibrated using the 14C calibration program CALIB, version 7.1.0 14ChronoCentre, QueensUniversityBelfast and the calibration curve IntCal13 [8].
